# Intranasal mesenchymal stem cell secretome administration markedly inhibits alcohol and nicotine self-administration and blocks relapse-intake: mechanism and translational options

**DOI:** 10.1186/s13287-019-1304-z

**Published:** 2019-07-08

**Authors:** María Elena Quintanilla, Fernando Ezquer, Paola Morales, Daniela Santapau, Pablo Berríos-Cárcamo, Marcelo Ezquer, Mario Herrera-Marschitz, Yedy Israel

**Affiliations:** 1 Molecular and Clinical Pharmacology Program, Institute of Biomedical Sciences, Santiago, Chile; 2 Department of Neuroscience, Faculty of Medicine, University of Chile, Santiago, Chile; 3 Centro de Medicina Regenerativa, Facultad de Medicina Clínica Alemana-Universidad del Desarrollo, Av. Las Condes 12438, Lo Barnechea, 7710162 Santiago, RM Chile; 4 Department of Pathology, Anatomy and Cell Biology, Thomas Jefferson University, Philadelphia, PA USA

**Keywords:** ROS, Neuroinflammation, Knockdown, GLT-1 antisense, Intranasal, Mesenchymal stem cells

## Abstract

**Background:**

Chronic consumption of most drugs of abuse leads to brain oxidative stress and neuroinflammation, which inhibit the glutamate transporter GLT-1, proposed to perpetuate drug intake. The present study aimed at inhibiting chronic ethanol and nicotine self-administration and relapse by the non-invasive intranasal administration of antioxidant and anti-inflammatory secretome generated by adipose tissue-derived activated mesenchymal stem cells. The anti-addiction mechanism of stem cell secretome is also addressed.

**Methods:**

Rats bred for their alcohol preference ingested alcohol chronically or were trained to self-administer nicotine. Secretome of human adipose tissue-derived activated mesenchymal stem cells was administered intranasally to animals, both (i) chronically consuming alcohol or nicotine and (ii) during a protracted deprivation before a drug re-access leading to relapse intake.

**Results:**

The intranasal administration of secretome derived from activated mesenchymal stem cells inhibited chronic self-administration of ethanol or nicotine by 85% and 75%, respectively. Secretome administration further inhibited by 85–90% the relapse “binge” intake that occurs after a protracted drug deprivation followed by a 60-min drug re-access. Secretome administration fully abolished the oxidative stress induced by chronic ethanol or nicotine self-administration, shown by the normalization of the hippocampal oxidized/reduced glutathione ratio, and the neuroinflammation determined by astrocyte and microglial immunofluorescence. Knockdown of the glutamate transporter GLT-1 by the intracerebral administration of an antisense oligonucleotide *fully* abolished the inhibitory effect of the secretome on ethanol and nicotine intake.

**Conclusions:**

The non-invasive intranasal administration of secretome generated by human adipose tissue-derived activated mesenchymal stem cells markedly inhibits alcohol and nicotine self-administration, an effect mediated by the glutamate GLT-1 transporter. Translational implications are envisioned.

**Electronic supplementary material:**

The online version of this article (10.1186/s13287-019-1304-z) contains supplementary material, which is available to authorized users.

## Background

Numerous preclinical and clinical studies have shown that oxidative stress and neuroinflammation are generated following chronic alcohol consumption or its administration [1–11]. It has been reported that ethanol intake, via the generation of acetaldehyde, weakens the tight junction intestinal barrier and promotes lipopolysaccharide influx from the gut into the portal circulation [12], leading to the increases in systemic TNF-α and the induction of neuroinflammation via the brain TNF-α receptor [13].

Heavy smoking often accompanies alcohol use disorders [14, 15]. Nicotine is the major addictive component of cigarette smoke and the prime culprit of the failure to quit smoking [16]. Chronic nicotine administration also leads to brain oxidative stress and activates NF-κB transcription, inducing neuroinflammation [17–20].

Recent studies [11] showed that rats that had ingested a nicotine solution (40 mg/l) for 2 months, under a free-choice paradigm versus water, display marked increases in brain oxidative stress, as seen by an increased oxidized/reduced glutathione (GSSG/GSH) hippocampal ratio. Nicotine is known to release dopamine into the nucleus accumbens, where high dopamine levels induce oxidative stress via DA auto-oxidation [21]. Further, the monoamine oxidase metabolism of dopamine generates hydrogen peroxide, a precursor to hydroxyl radicals [21, 22]. Nicotine also elevates both cytochrome P450 (CYP) 2A6 and CYP2E1, potent generators of oxygen radicals [23].

It has been shown that oxidative stress and neuroinflammation are tightly linked; TNF-α increases the generation of reactive oxygen species (ROS) via cytosolic and mitochondrial mechanisms [24–26]. In turn, ROS activates the NF-κB system partly by inactivating the NF-κB inhibitor IκB [27] allowing the NF-κB-mediated synthesis of TNF-α, thus creating a self-perpetuating neuroinflammation-oxidative stress vicious-like cycle.

Beyond the initial receptor-specific reinforcing effects of different drugs, the perpetuation of intake and the relapse of most drugs of abuse occur mainly by a general drug-induced cued-recall activation of the nucleus accumbens by glutamate released via hippocampal connection [28, 29]. In operant studies, chronic ethanol self-administration leads to a cued-induced elevation of extracellular glutamate in the nucleus accumbens [30], an elevation that is greatly increased if glutamate reuptake by the glial glutamate transporter GLT-1 is inefficient; this is also expected for nicotine [29]. Reactive oxygen species directly inhibit the glutamate GLT-1 transporter [31]; additionally, the ROS-generated lipoperoxidation product 4-hydroxynonenal (4HNE) forms adducts with the GLT-1 transporter, further inhibiting glutamate uptake by astrocytes [32, 33].

Mesenchymal stem cells (MSCs) are referred to as “guardians of inflammation” [34], being activated by pro-inflammatory signals to release anti-inflammatory cytokines and antioxidant molecules [8, 34]. Thus, MSCs have been proposed as potent therapeutic agents in the treatment of several conditions associated with neuroinflammation and oxidative stress [35]. Recent studies showed that the intranasal administration of MSC-derived exosomes (nanovesicles separated by ultracentrifugation from the secretome released from MSCs) to animals chronically ingesting ethanol temporally reverses neuroinflammation, lowers brain ROS levels, and inhibits alcohol intake [10]. However, the inhibitory effect of MSC exosomes on ethanol intake was considerably less effective and shorter acting than that reported [9] to follow the intravenous administration of intact MSCs, which after been activated release both exosomes plus soluble molecules, likely acting via a paracrine effect. Since whole secretome is expected to display effects more akin to MSC “pumps,” in the present study, we tested whether the non-invasive intranasal administration of the complete secretome derived from adipose tissue MSCs activated in vitro in pro-inflammatory conditions results in a strong therapeutic effect in inhibiting chronic ethanol intake and a longer action in preventing alcohol relapse binge drinking than that observed for MSC-derived exosomes [10].

Present studies also allowed us to determine the secretome effects on the self-administration of another drug of abuse, namely nicotine, thus testing the hypothesis that intake perpetuation of drugs of abuse (alcohol and nicotine) shares a common mechanism.

Studies presented determine if intranasally administered human adipose tissue-derived MSC secretome (i) inhibits chronic alcohol and nicotine intake and their relapse, (ii) normalizes chronic ethanol- and nicotine-induced hippocampal oxidative stress, and (iii) reverses the neuroinflammation induced by both drugs of abuse. (iv) Knockdown studies tested the hypothesis that the glutamate transporter GLT-1 mediates the inhibitory effect of MSC secretome on chronic alcohol and nicotine intake.

## Methods

### Animals

Two-month-old female Wistar-derived rats selectively bred as alcohol consumers (University of Chile Bibulous (UChB)) [11, 36] were used in the experiments. All animal procedures were approved by the Committee for Experiments with Laboratory Animals at the Medical Faculty of the University of Chile (Protocol CBA# 0994 FMUCH).

### Isolation, expansion, and characterization of human adipose tissue-derived MSCs

Human adipose tissue-derived MSCs were isolated from subcutaneous adipose tissue (abdominal region), obtained from liposuction aspirates of patients undergoing cosmetic liposuction at Clínica Alemana, Santiago, Chile, as previously described [37]. For all samples, a written informed consent was obtained. All protocols were approved by the Ethics Committee of Facultad de Medicina, Clínica Alemana-Universidad del Desarrollo. After two subcultures, cells were characterized according to their adipogenic and osteogenic differentiation potential, by the presence of putative hMSC markers (CD29, CD13, CD105, CD73, and CD90), and the absence of marker characteristics of other cell lineages (CD235a, CD31, and CD45) as previously described [37].

### Activation of human adipose tissue-derived MSCs and secretome generation

Human adipose tissue-derived MSCs (passage 3) at 70% of confluence were activated by incubation in minimal essential medium (α-MEM Gibco, Grand Island, NY) supplemented with 10% fetal bovine serum (HyClone, South Logan, UT) plus 10 ng/ml TNF-α and 15 ng/ml IFN-γ (R&D System, Minneapolis, MN) for 40 h. We and others [8, 38] have previously described that this activation strategy greatly improves the production of anti-inflammatory factors compared to non-activated MSCs. After the activation, the cells were washed three times with phosphate-buffered saline (PBS) and incubated for 48 h with α-MEM. After that, the culture media (secretome) were collected and centrifuged at 400*g* for 10 min to remove the whole cells. The supernatant was centrifuged again at 5000*g* for 10 min to remove cell debris. This process reduces the contamination of the secretome with proteins release by the rupture of the cells. Finally, the secretome was filtered in 0.22-μm filters.

Secretomes were concentrated 30 times (*v*/*v*) by 3 kDa cutoff filters (Millipore, Carrigtwohill CO). The concentrates were washed with 15 ml of PBS and re-concentrated again with the same filters. The protein concentration was determined by the BCA protein assay kit (Thermo Scientific, Waltham, MA), and the secretome was frozen at − 80 °C until use. As a control, secretome obtained from the same number of non-activated MSCs was also generated.

### Determination of antioxidant capacity of MSC secretome

The total antioxidant capacity of 2 μg of secretome protein obtained from activated adipose tissue-derived MSCs was determined by the Antioxidant Assay Kit (Cayman Chemical). The antioxidant system includes superoxide dismutase, catalase, glutathione peroxidase, macromolecules such as ferritin, and an array of small molecules including ascorbic acid, α-tocopherol, β-carotene, reduced glutathione, uric acid, and bilirubin. The assay relies on the ability of antioxidants in the sample to inhibit the oxidation of 2,2′-azino-di-3-ethylbenzthiazoline sulphonate. Thus, the combined antioxidant activities of all the constituents of the MSC secretome were assessed. Data were compared against the antioxidant capacity of 2 μg of secretome protein obtained from non-activated MSCs and 2 μg of human albumin.

### Quantification of IL-10 and TGF-B1 levels in MSC secretome

IL-10 and TGF-B1 were used as markers of anti-inflammatory activity. IL-10 and TGF-B1 abundance were evaluated in the secretome obtained from 1 × 10^6^ activated and 1 × 10^6^ non-activated MSCs using the human IL-10 High sensitivity ELISA Kit (Invitrogen) and TGF-B1 ELISA Kit (Life Technologies), respectively.

### Behavioral procedures

#### Voluntary alcohol consumption test: free choice drinking paradigm

Twenty-six female UChB rats were used. Only female rats were used because (unlike males) females do not increase their body weight during the long-term (3–4 months) voluntary alcohol or nicotine consumption, allowing a more stable alcohol or nicotine intake over time, when ethanol or nicotine intake are expressed as grams of ethanol/nicotine consumed per kilogram body weight. Additionally, for rats selectively bred for their high-ethanol intake, the Indianapolis female rats have a higher (HAD-2) or equal (HAD-1) ethanol intake than males [39]. For Sardinian high-ethanol intake bred rats, females show higher levels of ethanol intake than males [40]. Females of the UChB rat line show a 20% higher ethanol intake than males (unpublished). Oral alcohol intake (10% ethanol *v*/*v* prepared from absolute ethanol diluted in tap water) was allowed for 10–12 weeks in a free-choice drinking paradigm versus tap water. On the last week, animals were offered 10% ethanol, 20% ethanol, and water. Ethanol intake was expressed as grams of ethanol consumed per kilogram body weight per day.

#### Relapse-like alcohol drinking

Following over 13 weeks of continuous ethanol access, animals were deprived of ethanol solutions, but not water, for 14 days after which the animals were allowed re-access to 10% and 20% ethanol solutions for only 60 min as previously described [8]. Ethanol intake was expressed as grams of ethanol consumed per kilogram body weight per 60 min.

#### Voluntary nicotine consumption and relapse-like nicotine self-administration

Twenty-six female UChB rats were allowed to self-administer nicotine solutions as described by Quintanilla et al. [11]. Essentially, rats were pre-treated for 14 days with a daily intraperitoneal dose (0.5 mg/kg) of nicotine hydrogen tartrate (Sigma-Aldrich, St. Louis, MO) dissolved in saline [17]. On day 15, nicotine intraperitoneal injections were discontinued, and rats were offered a free choice between ingesting increasing concentration of nicotine solutions (5 to 40 mg/l nicotine dissolved in water) or water. The nicotine solution was kept constant (24 h/day) at 40 mg/l for the last 15 weeks. Nicotine intake was expressed as milligrams of nicotine consumed per kilogram body weight per day. The development of the nicotine self-administration model is shown in Additional file 1: Figure S1. Following 22 weeks of continuous access to nicotine and water, animals were deprived of nicotine for 13 days and were thereafter allowed re-access to a 40-mg/l nicotine solution and water for only 60 min [11]. Nicotine intake upon re-access was expressed as milligrams of nicotine consumed per kilogram body weight per 60 min.

#### Intranasal administration of MSC-derived secretome

Rats were anesthetized with chloral hydrate (280 mg/kg, i.p.) and placed in the supine position. Twenty microliters of secretome solution was administered intranasally as drops delivered from a small pipette tip every 5 min (four times in each nostril) into alternative sides of the nasal cavity for a total of 20 min. A total volume of 160 μl of secretome containing 25 μg of total proteins derived from 1 × 10^6^ adipose tissue-derived activated MSCs was delivered into the nasal cavity. Control animals received 160 μl of saline by the same administration scheme [10].

#### Determination of chronic ethanol and nicotine intake and relapse following the intranasal MSC secretome administration

Two groups of rats were used. The first group of rats (*n* = 10) had ingested ethanol under the free-choice condition for 74 days, while the second group (*n* = 10) had ingested nicotine under the free-choice condition for 138 days. Each group was divided into two subgroups that received a weekly intranasal dose of secretome or vehicle (saline) for 5 weeks. The first three doses were administered while the rats were under the free choice of chronic ethanol or chronic nicotine intake, while the last two doses were administered while the animals were under a 13- to 14-day period of nicotine or ethanol deprivation. Three days after the last intranasal secretome or vehicle administration, re-access to 10% and 20% ethanol or to 40 mg/l nicotine and water for a 60-min period was allowed. Immediately after re-access to the ethanol or nicotine solutions, the animals were anesthetized with chloral hydrate (280 mg/kg, i.p.) and blood samples were obtained for the assessment of the blood ethanol or plasma cotinine, the major metabolite of nicotine. Then, the animals were perfused intracardially with 100 ml of PBS (pH 7.4) and euthanized to obtain brain samples for the determination of hippocampus GSSG/GSH ratio and glial reactivity.

#### Blood ethanol determination

Blood samples (0.1 ml) were collected without anticoagulant from the tip of the tail under moderate sedation with acepromazine (1 mg/kg, i.p.). The samples were analyzed by headspace gas chromatography (Perkin Elmer SRI 8610) as previously described [8].

#### Plasma cotinine determination

Immediately following a 60-min re-access to nicotine (40 mg/l) after the 13-day nicotine deprivation period, rats were anesthetized with chloral hydrate (280 mg/kg, i.p.) and blood (1.5 ml) samples were collected by cardiac puncture. Cotinine level was determined in the plasma by ELISA using the Cotinine ELISA Kit (MyBiosource, San Diego, CA).

#### GSSG/GSH ratio determination

The hippocampi were extracted and mixed with three volumes of ice-cold potassium buffer (0.1 M) containing 5 mM EDTA, pH 7.4. Reduced glutathione (GSH) and oxidized glutathione disulfide (GSSG) content were determined as described previously [8].

#### Tissue preparation and glial immunohistochemistry

Preparation of brain tissue for immunohistochemistry against glial fibrillary acidic protein (GFAP)-positive astrocytes and ionized calcium-binding adaptor molecule 1 (Iba-1) for microglial cells was performed according to Morales et al. [41], Perez-Lobos et al. [42], and Ezquer et al. [10]. The samples were examined by confocal microscopy (Olympus-fv10i). An unbiased stereological approach was used to analyze GFAP-positive astrocytes, quantifying the total length and thickness of primary processes and density of Iba-1-positive microglial cells in the hippocampus, since this glia has a significant role in the development and maintenance of drug addiction [1, 43, 44].

#### Effect of glutamate transporter GLT-1 knockdown on chronic ethanol or nicotine intake following the intranasal administration of MSC-derived secretome

Two groups of rats were used: rats of the alcohol group (*n* = 16) that had ingested ethanol for 88 days and rats of the nicotine group (*n* = 16) that had ingested nicotine for 153 days. All animals were pre-anesthetized with chloral hydrate (280 mg/kg, i.p.), maintained under anesthesia with a mixture of air and isofluorane, and mounted on a stereotaxic frame to be injected with the oligonucleotides into the left lateral ventricle (AP 0, − 1.2, − 3.8 mm), according to Paxinos and Watson [45]. The alcohol and nicotine groups were divided into four subgroups (*n* = 4) that were injected intracerebro-ventricularly (icv) with: group #1, 10 μl of GLT-1 oligo antisense (17 nmol); group #2, 10 μl of control oligo antisense (17 nmol); group #3, 10 μl of vehicle (saline); or group #4, 10 μl of vehicle (saline). The sequences of antisense oligonucleotide anti-GLT-1 and the control oligonucleotide (Integrated DNA Technologies, NC) were those described by Reissner et al. [46]. However, phosphorothioate rather than morpholino moieties constituted the backbone of the molecule administered. Thereafter, the animals were returned to their cages and allowed access to ethanol or nicotine solutions and water under free choice. Twenty-four hours after the intracerebral administration rats of group #1, group #2, and group #3 received intranasally 160 μl of secretome (25 μg of secretome proteins, derived from 1 × 10^6^ activated MSCs), while rats of group #4 received 160 μl of vehicle, all under chloral hydrate (280 mg/kg, i.p.) anesthesia. After the intranasal administration, the animals were allowed to continue drinking alcohol or nicotine under free choice, for 48 additional hours.

#### Statistical analyses

Statistics were performed using GraphPad Prism software (San Diego, CA). For behavioral experiments, two-way ANOVA (treatment × day) with or without repeated measures followed by Bonferroni’s or Tukey’s post-hoc tests were used. For GSSG/GSH ratio, the total length and thickness of primary astrocytic processes and microglia density, one-way ANOVA followed by Tukey or Bonferroni post-hoc test were used for testing the differences among the groups. When only two groups were compared, statistical significance was determined by Student’s *t* test. Significance was set at *p* < 0.05, and all data are presented as mean ± SEM. Full statistical analyses of each figure are presented in the legend of the figure.

## Results

### Intranasal administration of secretome derived from adipose tissue-derived activated MSCs inhibits chronic alcohol and chronic nicotine intake and blocks post-deprivation relapse

It has been previously reported that human MSCs can produce a broad range of anti-inflammatory and antioxidant factors, and this paracrine potential can be boosted by activating the cells by incubation with pro-inflammatory cytokines, enhancing their therapeutic efficacy [8]. Initially, as a proof of concept, we evaluated if the intranasal administration of secretome derived from *non-activated* MSCs could inhibit chronic ethanol intake. Additional file 2: Figure S2A shows that secretome obtained from 1 × 10^6^ adipose tissue-derived MSCs that were *not activated* only marginally and transiently inhibited chronic alcohol intake and did not prevent a relapse binge drinking (i.e., blood alcohol concentration BAC > 80 mg/dl) (Additional file 2: Figure S2B), suggesting (vide infra) that the activation of MSCs is needed to improve the anti-addictive effect of the secretome.

**Fig. 1:**
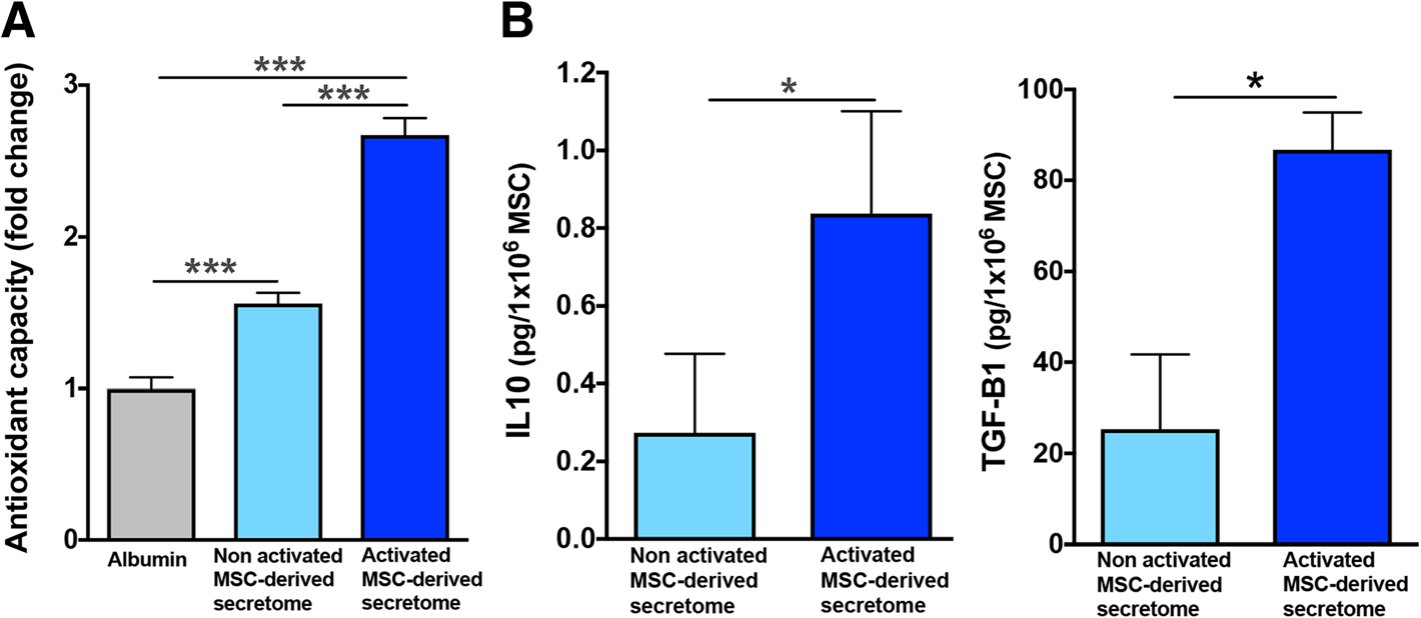
Secretome derived from human adipose tissue-derived MSCs activated with TNF-α and IFN-γ displays an enhanced antioxidant and anti-inflammatory activity. **a** Secretome derived from non-activated MSCs (light blue bar) shows a lower antioxidant capacity, compared with that of activated MSC-derived secretome (blue bar). Antioxidant capacity of MSC-derived secretome was also compared to the antioxidant capacity of human albumin (gray bar) (one-way ANOVA *F*_2,9_ = 92.62, *p* < 0.0001. Tukey post-hoc: *p* < 0.001 non-activated MSC secretome versus albumin; *p* < 0.0001 activated MSC secretome versus albumin and versus non-activated MSC secretome; *n* = 4 samples per group). **b** Secretome derived from activated MSCs shows a threefold increase in anti-inflammatory IL-10 and TGF-B1 levels compared with secretome derived from non-activated MSCs (*t* test; *p* < 0.05, *n* = 4 samples per group)

Adipose tissue-derived MSCs were activated by incubation with 10 ng/ml TNF-α and 15 ng/ml IFN-γ for 40 h. Secretome generated following the above pro-inflammatory activation of MSCs markedly increased its antioxidant capacity (Fig. 1a) and its anti-inflammatory activity, determined by increased in IL-10 and TGF-B1 levels (Fig. 1b) compared to the secretome derived from non-activated MSCs. These results are in line with previous reports indicating that pro-inflammatory activation of MSCs greatly improved the production and secretion of several anti-inflammatory factors [8, 38].

**Fig. 2:**
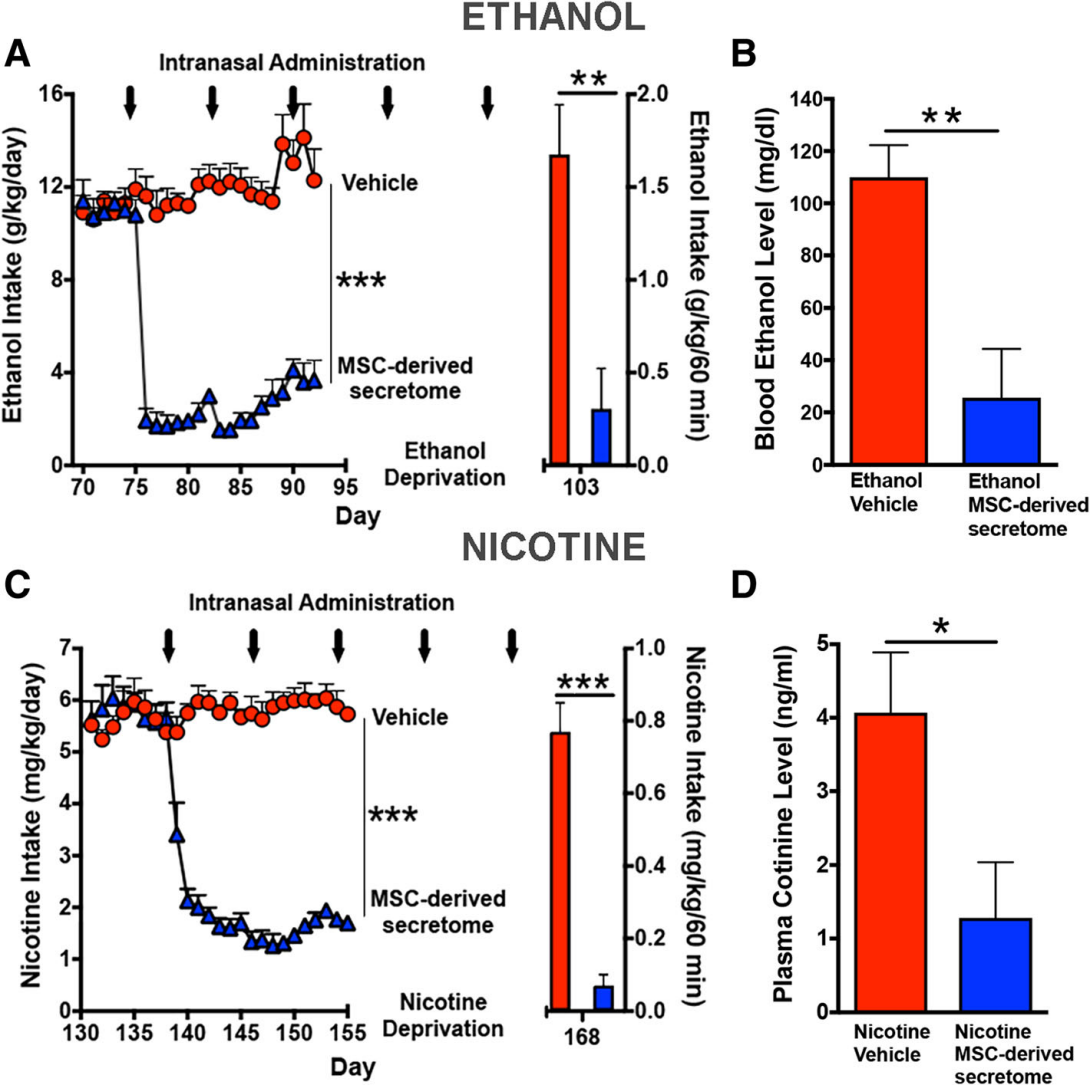
Intranasal administration of secretome derived from adipose tissue-derived activated MSCs inhibits chronic ethanol and chronic nicotine intake and blocks post-deprivation binge relapse. Ethanol intake (**a**, left): red circles show mean ± SEM daily ethanol intake of vehicle intranasal-treated rats (average intake 11.4 ± 0.3 g ethanol/kg body weight; *n* = 5) that were allowed free-choice access to ethanol (10% and 20% *v*/*v*) and water. Arrows indicate the weekly intranasal administration of MSC secretome (160 μl of saline containing 25 μg protein derived from 1 × 10^6^ activated MSCs) or 160 μl of vehicle. Two-way ANOVA (treatment × day) of ethanol intake following 3 intranasal doses of MSC secretome (blue triangles) indicates significant effect of treatment (*F*_1,184_ = 1179, *p* < 0.0001), day (*F*_22,184_ = 13.97, *p* < 0.0001), and significant interaction (*F*_treatment × day 22,184_ = 18.58, *p* < 0.001) compared with control rats receiving vehicle (red circles). Bonferroni post-hoc analysis revealed that secretome treatment inhibited ethanol intake (85%) during the 17 days recorded versus vehicle-treated control (*p* < 0.001; *n* = 5 per group). **a** (right) Red bars show ethanol intake after 2 weeks of ethanol deprivation followed by a 60-min period of 10% and 20% ethanol re-access. Rats treated previously with five intranasal secretome doses (blue bar) ingested a significantly lower amount of ethanol (average intake, 0.304 ± 0.22 g ethanol/kg/60 min) than vehicle control animals (red bar, average intake, 1.60 ± 0.27 g ethanol/kg/60 min) (two-tailed *t* test 3.936, *p* < 0.001; *n* = 5 rats/group). **b** Blood ethanol levels attained immediately after the 60-min ethanol re-access were significantly lower in rats treated with five MSC secretome doses (blue bar) (25.6 ± 18.7 mg/dl, mean ± SEM) than in vehicle control animals (red bar; 110 ± 12 mg/dl) (two-tailed *t* test = 3.77; *p* < 0.005; *n* = 5 rats/group). Nicotine intake (**c**, left): red circles show the mean (± SEM) daily nicotine intake of vehicle intranasal-treated rats (average 5.6 ± 0.06 mg nicotine/kg; *n* = 5) that were allowed free-choice access to nicotine (40 mg/l) and water. Two-way ANOVA (treatment × day) of nicotine intake data obtained following 3 intranasal MSC secretome doses (blue triangles) shows a significant effect of treatment (*F*_1,17_ = 1232, *p* < 0.0001), day *F*_24,175_ = 22.55, *p* < 0.0001) and significant interaction (*F*_treatment × day 24,175_ = 28.92, *p* < 0.0001) compared with control rats receiving vehicle (red circles). Bonferroni post-hoc test revealed that intranasal secretome induced significant inhibition of nicotine intake (75%) during the 17 days studied versus vehicle-treated rats (*p* < 0.001; *n* = 5 rats per group). **c** (right) Bars show the mean 60-min nicotine consumption upon the nicotine re-access relapse which followed 13 days of nicotine deprivation. Rats treated previously with five intranasal MSC secretome doses (blue bar) ingested a significantly lower amount of nicotine (0.07 ± 0.03 mg/60 min; mean ± SEM) than vehicle control rats (red bar, 0.77 ± 0.08 mg/60 min) (two-tailed *t* test = 8.193; *p* < 0.001; *n* = 5 rats/group). **d** Blue bar shows that plasma cotinine levels, the main nicotine metabolite, determined immediately after the 60-min nicotine re-access were significantly lower after five intranasal secretome doses (1.28 ± 0.76 ng/ml) than in the vehicle-treated animals (red bar, 4.07 ± 0.82 ng/ml) (two-tailed *t* test = 2.438; *p* < 0.04; *n* = 5 rats/group)

**Fig. 3:**
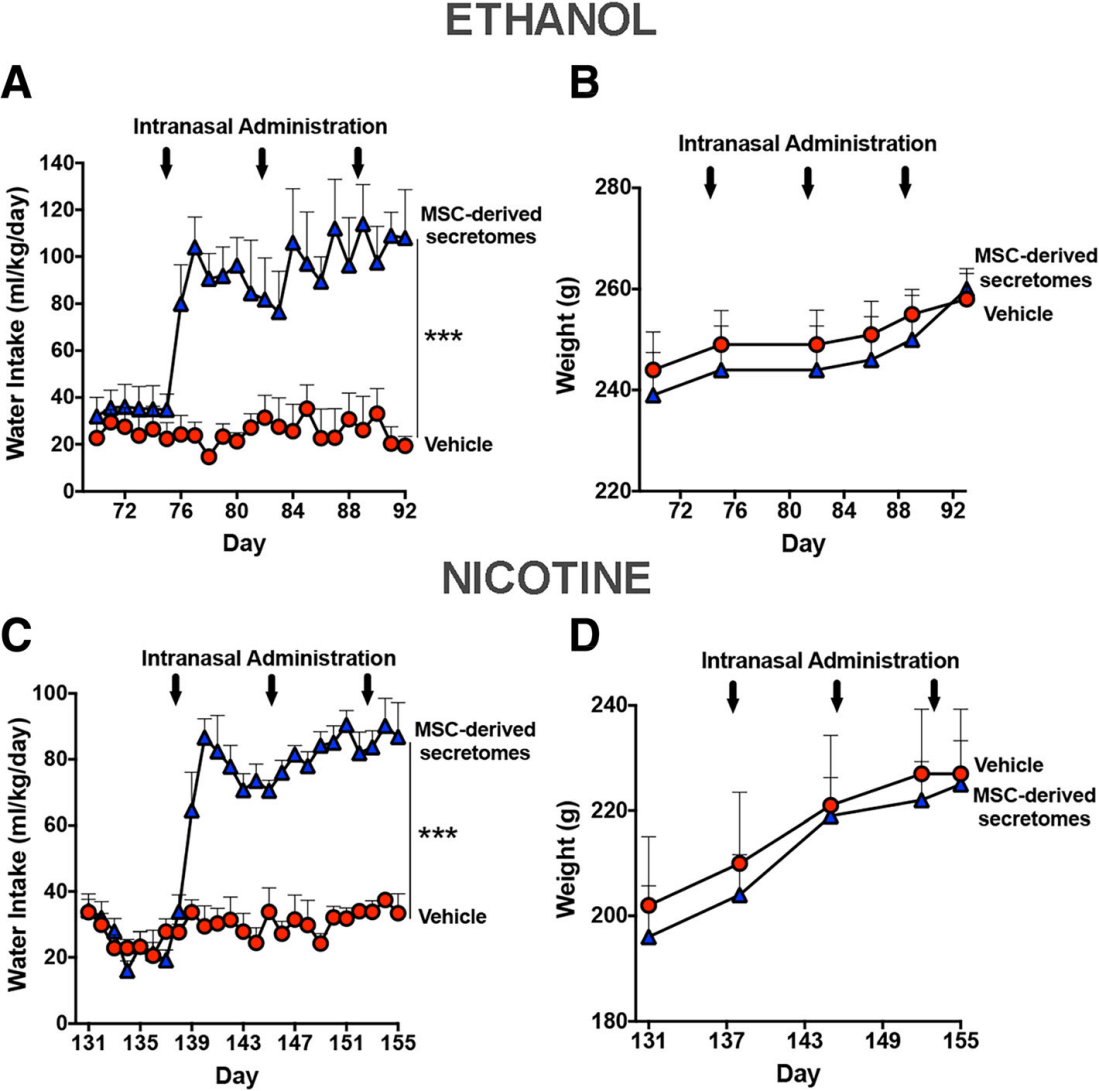
Secretome administered at weekly intervals to chronical ethanol- and nicotine-ingesting rats led to increases in water intake while not affecting body weight gain. **a** Three intranasal doses of secretome to chronic ethanol-drinking rats, that had resulted in a marked inhibition of ethanol intake (shown in Fig. 2a, left), induced, in the same animals, a significant increase in water intake (blue triangles) (*F*_1,161_ = 189.7, *p* < 0.001), significant effect of day (*F*_22,161_ = 2.34, *p* < 0.001), and significant interaction (*F*_22,161_ = 2.45, *p* < 0.001) versus control rats treated with vehicle (red circles). Bonferroni post-hoc analysis revealed that the intranasal administration of secretome significantly increases water intake during the period of secretome treatment versus control rats treated with vehicle (*p* < 0.001). **b** Secretome treatment did not affect the animal’s body weight gain (*F*_1,48_ = 0.83, *p =* 0.36; N.S.). **c** Three intranasal doses of secretome to chronic nicotine drinking rats, that had produced a marked inhibition of nicotine intake (shown in Fig. 2c,left), induced, in the same animals, a significant increase in water intake (blue triangles) (*F*_1,175_ = 443.2, *p* < 0.001), significant effect of day (*F*_24,175_ = 13.87, *p* < 0.001), and significant interaction (*F*_24,175_ = 9.42, *p* < 0.001) versus control rats treated with vehicle (red circles). Bonferroni post-hoc revealed that the intranasal administration of secretome significantly increases water intake during the 17 days of secretome treatment versus control rats treated with vehicle (*p* < 0.001). These findings suggest that the increase in water intake is a compensatory mechanism for the decrease in drug intake, which is keeping the total amount of body fluid intake. **d** Secretome treatment did not affect the animal’s body weight gain (*F*_1,35_ = 0.41, *p =* 0.52; N.S)

Figure 2 shows that the intranasal administration of secretome derived from 1 × 10^6^ activated MSCs markedly inhibited both chronic ethanol (Fig. 2a, left) and chronic nicotine (Fig. 2c, left) self-administration. A single intranasal administration of secretome derived from activated MSCs inhibited ethanol self-administration by 85% (*p* < 0.001) and nicotine self-administration by 75% (*p* < 0.001) versus their respective vehicle (saline) controls. Similar inhibitory effects were observed following three intranasal administrations of MSC secretome delivered at weekly intervals. A 14- or 13-day deprivation of ethanol or nicotine, respectively, imposed to animals previously allowed free chronic intake of ethanol or nicotine followed by a 60-min re-access to the ethanol (10% and 20%) or nicotine (40 mg/l) resulted in elevated relapse intakes of both alcohol and nicotine. Rats that received intranasal MSC secretome showed an 85% (*p* < 0.001) reduction of ethanol intake and a 90% (*p* < 0.001) reduction of nicotine intake versus their corresponding intranasal vehicle (saline) controls (Figs. 2a, c; right). For the vehicle-treated animals, blood alcohol levels (110 ± 12 mg/dl) determined immediately after the 60-min ethanol re-access exceeded those considered “binge drinking” (> 80 mg/dl) in humans, while in secretome-treated animals, blood ethanol levels were 25.6 ± 18.7 mg/dl (Fig. 2b¸ *p* < 0.005). Similarly, for the chronic nicotine group, blood cotinine levels of animals that received secretome derived from activated MSCs were 70–75% lower (*p* < 0.04) after re-access than those of animals that had received vehicle (4.07 ± 0.82 ng/ml) (Fig. 2D). For both the alcohol and nicotine post-deprivation and re-access studies, the last intranasal dose of secretome was administered three days prior to the 60-min drug re-access. Figure 3 shows that the reduction of both chronic ethanol and nicotine intakes (shown in Fig. 2) induced by the intranasal administration of secretome derived from activated MSCs were compensated by the increases in water intake (*p* < 0.001), thus normalizing the animal’s water homeostasis (Fig. 3a, c). Further, animal growth, determined by the weight gained during the treatment period, was not affected by MSC secretome administration (Fig. 3b, d).

### Intranasal administration of secretome derived from adipose tissue-derived activated MSCs reduced hippocampus oxidative stress induced by chronic ethanol and chronic nicotine intake

**Fig. 4:**
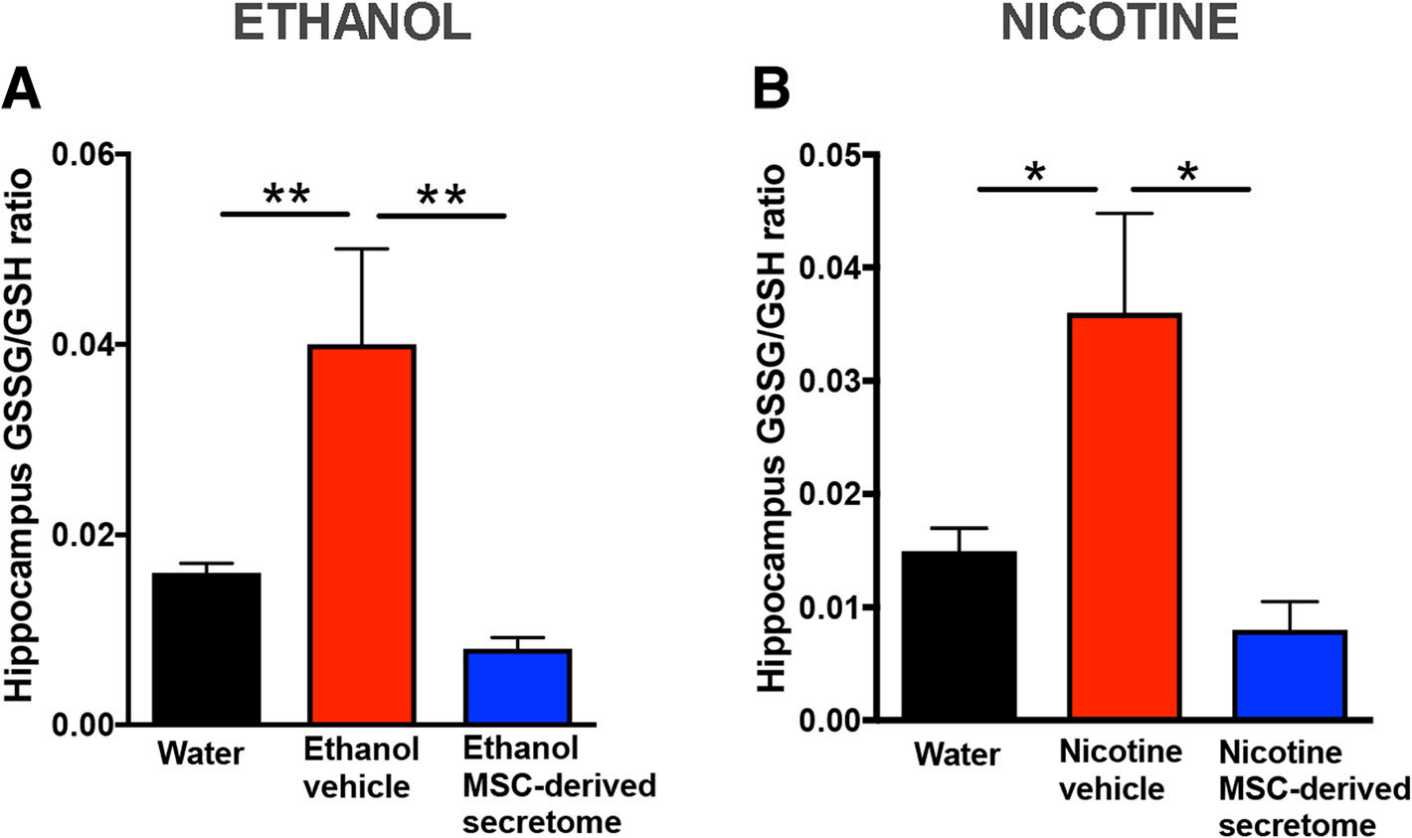
Intranasal administration of adipose tissue-derived MSC secretome reduced hippocampus oxidative stress (assessed by GSSG/GSH ratio) induced by chronic ethanol and chronic nicotine intake. The hippocampi were obtained from rats chronically drinking ethanol or nicotine immediately after 60 min of re-exposure to ethanol or nicotine solution, following a 2-week deprivation of ethanol or nicotine. **a** Chronic ethanol drinking rats treated with vehicle displayed an increased GSSG/GSH ratio (red bar) versus ethanol-naïve rats drinking only water (black bar). Administration of five intranasal doses of MSC secretome, administered at weekly intervals to chronic ethanol drinking rats, normalized the GSSG/GSH ratio (blue bar) (one-way ANOVA: *F*_2,12_ = 11.0, *p* < 0.001; Bonferroni post-hoc: *p* < 0.01, water drinking versus ethanol-vehicle; *p* < 0.01, ethanol-secretome versus ethanol-vehicle; *n* = 5 per group). **b** Chronic nicotine-drinking rats treated with vehicle displayed an increased GSSG/GSH ratio (red bar) versus naïve rats drinking only water (black bar). Administration of five intranasal doses of secretome, administered at weekly intervals to chronic nicotine drinking rats, resulted in the normalization of the GSSG/GSH ratio (blue bar) (one-way ANOVA: *F*_2,9_ = 7.26, *p* < 0.01; Bonferroni post-hoc analysis: *P* < 0.02, water drinking control rats versus nicotine-vehicle; *p* < 0.01, nicotine-secretome versus nicotine-vehicle; *n* = 5 per group)

Increase in brain oxidative stress has been reported to follow both chronic ethanol and nicotine intakes and may perpetuate drug consumption [3, 20]. Figure 4 shows that the hippocampal GSSG/GSH ratio (a marker of oxidative stress) determined immediately after the 60-min drug re-access was two- to threefold higher in animals that had chronically ingested ethanol (*p* < 0.01) or nicotine (*p* < 0.02) versus that in naïve water-consuming animals. The intranasal administration of MSC secretome (Fig. 4a, b) fully reversed the elevated GSSG/GSH values both for chronic ethanol- (*p* < 0.01) or chronic nicotine-consuming (*p* < 0.01) animals. Noteworthy, the GSSG/GSH elevations seen in ethanol and nicotine vehicle-treated animals occur despite a nicotine or ethanol deprivation lasting 13 to 14 days (in line with a self-perpetuation of oxidative stress after a prolonged drug deprivation).

### Intranasal administration of secretome derived from adipose tissue-derived activated MSCs normalized both the increased astrocyte reactivity and the increased microglial density induced by chronic ethanol and chronic nicotine intake

**Fig. 5:**
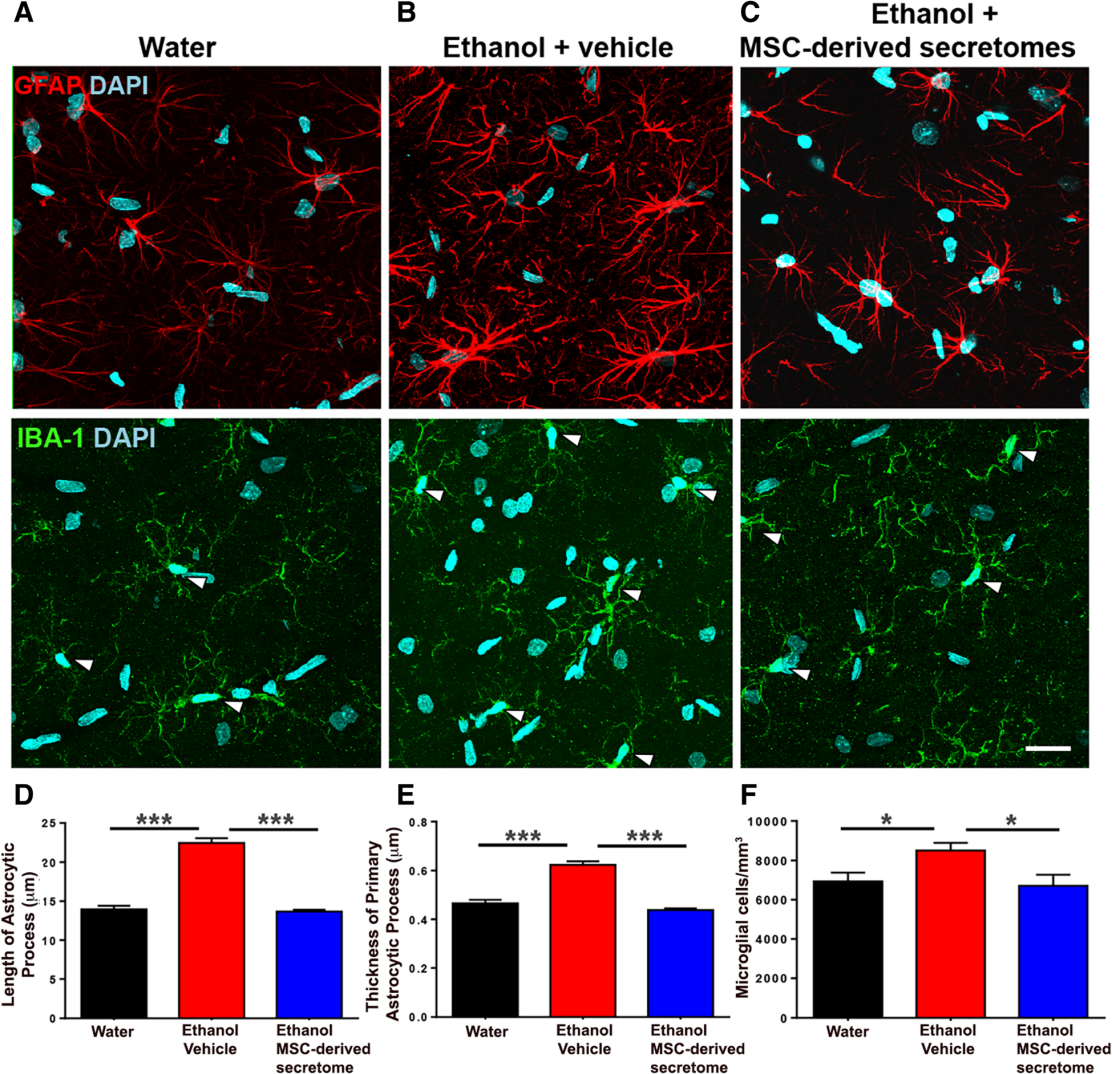
Intranasal administration of adipose tissue-derived MSC secretome normalized both the increased astrocyte reactivity and the increased microglial density induced by chronic ethanol intake. Representative confocal microphotographs of hippocampal astrocyte GFAP immunoreactivity (red, top) and microglial density (Iba-1; green, shown by arrows, center) immunoreactivity counterstained with DAPI (blue, nuclear marker) (scale bar 25 μm). Chronic ethanol-drinking rats treated with vehicle displayed a marked increase in the length and thickness of astrocyte processes (**b**, top) and microglial density (**b**, center) versus rats drinking only water (**a**, top and center). Administration of five intranasal doses of secretome, administered at weekly intervals (blue bar), to chronic ethanol drinking rats normalized the length and thickness of astrocytic process and microglial density (**c**, top and center). One-way ANOVA of ethanol-drinking vehicle-treated rats versus water-drinking rats respect to (i) the length of astrocyte processes (**d**); *F*_2, 1984_ = 153.6, *p <* 0.0001; Tukey post-hoc: *p* < 0.001; (ii) the thickness of primary processes (**e**); *F*_2, 727_ = 93.04, *p* < 0.0001; Tukey post-hoc: *p* < 0.001; and (iii) microglial cell density/mm^3^ (**f**); *F*_2, 56_ = 5.246, *p <* 0.05; Tukey post-hoc: *p* < 0.001. One-way ANOVA ethanol-drinking rats secretome-treated versus ethanol-drinking rats vehicle treated respect to (i) the length of astrocyte processes (**d**); *F*_2, 1984_ = 153.6, *p* < 0.001; Tukey post-hoc: *p* < 0.001; (ii) the thickness of primary process (**e**); *F*_2, 727_ = 93.04, *p* < 0.001; Tukey post-hoc: *p* < 0.001; and (iii) microglial cell density/mm^3^ (**f**); *F*_2,56_ = 5.246; *p* < 0.01; Tukey post-hoc: *p* < 0.05

As indicated earlier, oxidative stress and neuroinflammation are tightly linked [26]. Since the latter cannot be readily gauged by assessing the levels of pro- and anti-inflammatory cytokines, present studies addressed the combined end point, namely inflammation-associated glial reactivity (morphological changes in astrocytes and microglial density). As seen in Fig. 5a and b (top), chronic ethanol intake markedly increased both the length and thickness of hippocampal astrocyte processes (GFAP immunofluorescence; red) versus those of naïve rats, data quantified in Fig. 5d and e (*p* < 0.001). Intranasal MSC secretome administration fully reversed the ethanol-induced changes in length (Fig. 5b, c (top), d; *p* < 0.001) and thickness (Fig. 5b, c (top), e; *p* < 0.001) of astrocytic processes. Chronic ethanol intake led to moderate increases in microglial density (number/area) (IBA-1 immunofluorescence; green) (Fig. 5a, b (center); *p* < 0.05), changes that were suppressed (Fig. 5f; *p* < 0.05) by the intranasal MSC secretome administration.

**Fig. 6:**
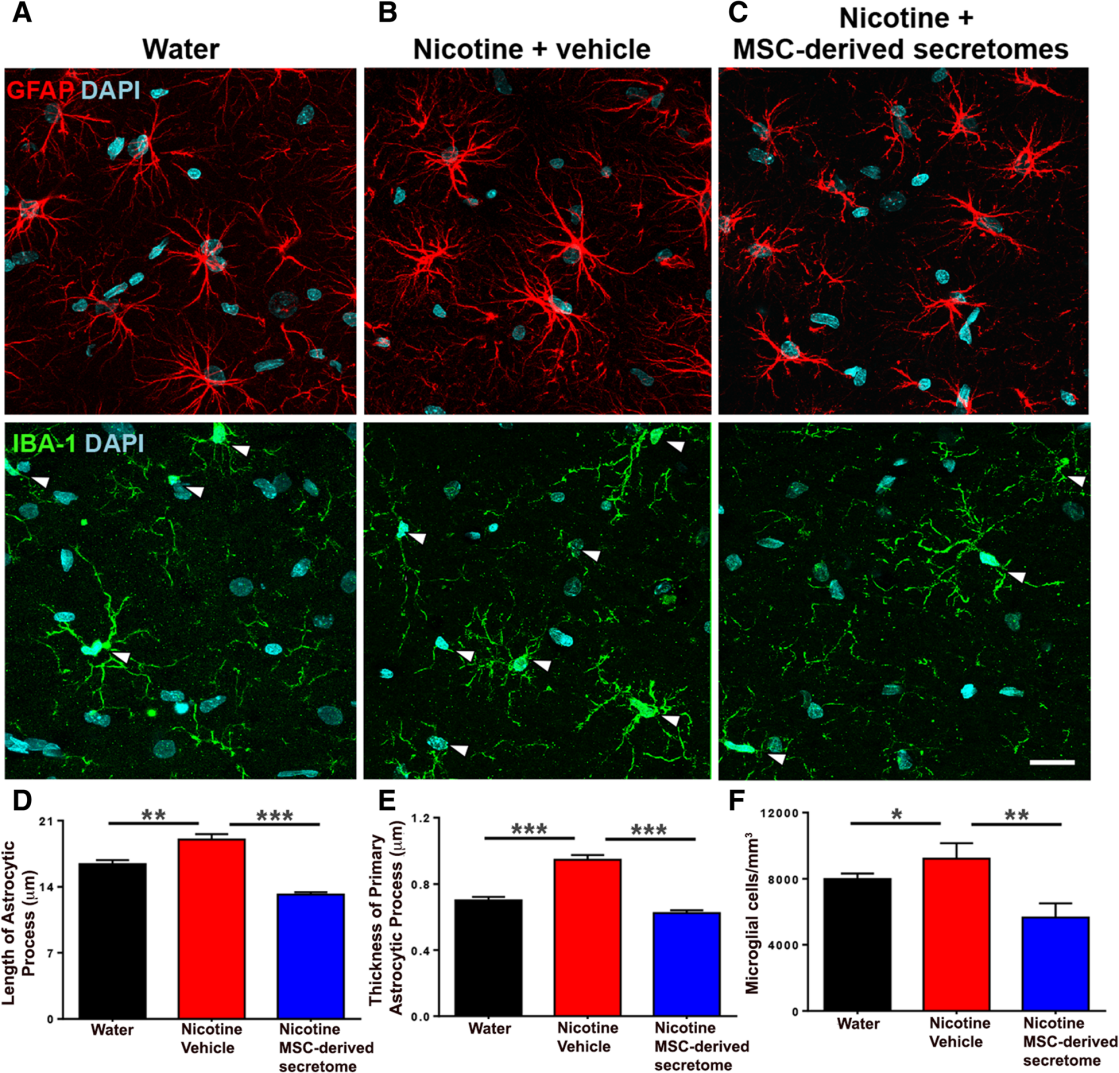
Intranasal administration of adipose tissue-derived MSC secretome normalized increases in astrocyte reactivity and in microglial density induced by chronic nicotine intake. Representative confocal microphotographs of hippocampal astrocyte GFAP immunoreactivity (red, top) and microglial density (Iba-1; green, shown by arrows, center) immunoreactivity counterstained with DAPI (blue, nuclear marker) (scale bar, 25 μm). Chronic nicotine-drinking rats treated with vehicle displayed a marked increase in the length and thickness of astrocyte processes (**b**, top) and microglial density (**b**, center) versus rats drinking only water (**a**, top and center). Administration of five intranasal doses of secretome, administered at weekly intervals (blue bar) to chronic nicotine-drinking rats, normalized the length and thickness of astrocytic process and microglial density (**c**, top and center). One-way ANOVA of nicotine-drinking vehicle-treated rats versus water-drinking rats respect to (i) the length of astrocyte processes (**d**); *F*_2, 1858_ = 46.19, *p* < 0.0001; Tukey post-hoc: *p* < 0.001; (ii) the thickness of primary processes (**e**); *F*_2, 727_ = 93.04, *p* < 0.0001; Tukey post-hoc: *p* < 0.0001; and (iii) microglial cell density/mm^3^ (**f**); *F*_2,443_ = 47.28, *p* < 0.01; Tukey post-hoc: *p* < 0.0001. One-way ANOVA nicotine-drinking rats secretome-treated versus nicotine-drinking vehicle-treated rats respect to (i) the length of astrocyte processes (**d**); *F*_2, 1858_ = 46.19, *p* < 0.001; Tukey post-hoc: *p* < 0.0001; (ii) the thickness of primary process (**e**); *F*_2, 443_ = 47.28, *p* < 0.0001; Tukey post-hoc: *p* < 0.0001; and (iii) microglial cell density/mm^3^ (**f**); *F*_2,52_ = 8.855; *p* < 0.01; Tukey post-hoc: *p* < 0.01

As shown in Fig. 6a and b (top), chronic nicotine intake also increased both the length and thickness of hippocampal astrocyte processes (GFAP red immunofluorescence) versus those of naïve water-consuming animals (Fig. 6d, e; *p* < 0.005). The nicotine-induced changes in astrocyte length (Fig. 6d) and thickness (Fig. 6e) were fully reversed (*p* < 0.001) following the intranasal MSC secretome administration. Figure 6a and b (center) show that chronic nicotine intake increased microglial density (cell number/area) (Fig. 6f; *p* < 0.05), changes that were also suppressed by intranasal MSC secretome administration (Fig. 6f; *p* < 0.01).

### A single intranasal dose of secretome derived from adipose tissue-derived activated MSCs inhibited ethanol and nicotine intake and reduced the hippocampal oxidative stress induced by chronic ethanol or nicotine intake

**Fig. 7:**
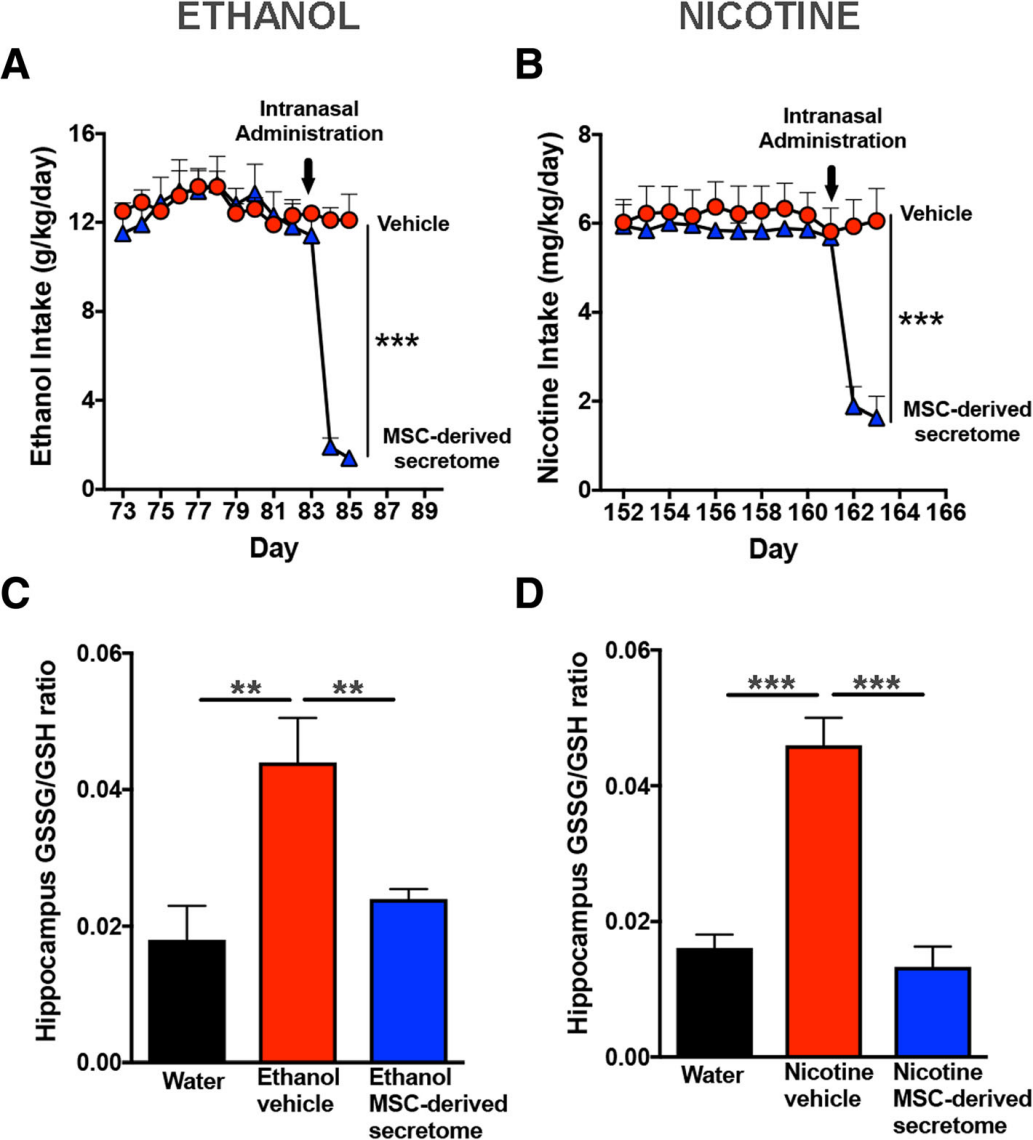
A single intranasal dose of adipose tissue-derived MSC secretome inhibited ethanol and nicotine intake and reduced the hippocampal oxidative stress (assessed by GSSG/GSH ratio) induced by chronic ethanol or nicotine intake. The hippocampi were extracted from rats chronically drinking alcohol or nicotine solutions 24 h a day, without deprivation periods. **a** Intranasal administration of one dose of MSC-derived secretome inhibited (85%) chronic ethanol intake within 48 h after the administration (two-tailed Student *t* test = 10.04, *p* < 0.001). **b** A single intranasal dose of MSC-derived secretome inhibited (75%) chronic nicotine intake (two-tailed Student *t* test = 5.06, *p* < 0.001) within 48 h after secretome administration. **c** Chronic ethanol-drinking rats treated with vehicle displayed an increased GSSG/GSH ratio (red bar) versus ethanol-naïve rats drinking only water (black bar). A single intranasal dose of secretome administered to ethanol drinking rats resulted in a full normalization of the GSSG/GSH ratio (blue bar) (one-way ANOVA: *F*_2,9_ = 8.0, *p* < 0.01; Bonferroni post-hoc: *p* < 0.01, water drinking versus ethanol-vehicle; *p* < 0.02, ethanol-secretome versus ethanol-vehicle; *n* = 5 per group). **d** Chronic nicotine-drinking rats treated with vehicle displayed an increased GSSG/GSH ratio (red bar) versus naïve rats drinking only water (black bar). A single intranasal dose of secretome administered to nicotine-drinking rats resulted in a full normalization of the GSSG/GSH ratio (blue bar, one-way ANOVA: *F*_2,9_ = 34.0, *p* < 0.0001; Bonferroni post-hoc: *p* < 0.01, water drinking versus ethanol-vehicle; *p* < 0.02, ethanol-secretome versus ethanol-vehicle; *n* = 5 per group)

It is noted that in the above study, in which secretome derived from adipose tissue-derived activated MSCs was shown to abolish ethanol- and nicotine-induced increases in hippocampal oxidative stress (GSSG/GSH ratio), the animals were drug deprived for nearly 2 weeks prior to being allowed a 60-min re-access to ethanol or nicotine solutions, which led to “binge-like” relapse intakes. To determine if chronic ethanol and nicotine intake per se, rather than a “binge-like” relapse intake, leads to increases in hippocampal oxidative (GSSH/GHS) stress and is responsible for the secretome effect, a separate study was conducted in which secretome was administered to chronical ethanol- and nicotine-consuming animals that were *not* subjected to a drug deprivation period nor to the relapse condition. In such study, marked inhibitions of chronic ethanol intake (− 85%; *p* < 0.001) and chronic nicotine intake (− 75%, *p* < 0.001) were again observed following a single intranasal administration of MSC secretome (Fig. 7a, b). Also seen were marked (two- to threefold) increases in hippocampal GSSG/GSH ratios (ethanol, *p* < 0.01; nicotine, *p* < 0.001), which were fully normalized (*p* < 0.02 and *p* < 0.001, respectively) by a single dose of secretome derived from activated MSCs (Fig. 7c, d). Thus, chronic ethanol or nicotine intake per se, rather than a high drug intake upon relapse, leads to increases in hippocampal oxidative (GSSH/GHS) stress, which are fully normalized by secretome administration.

### Knockdown of GLT-1 prevented the inhibition of ethanol and nicotine intake induced by intranasal administration of secretome derived from adipose tissue-derived activated MSCs

**Fig. 8:**
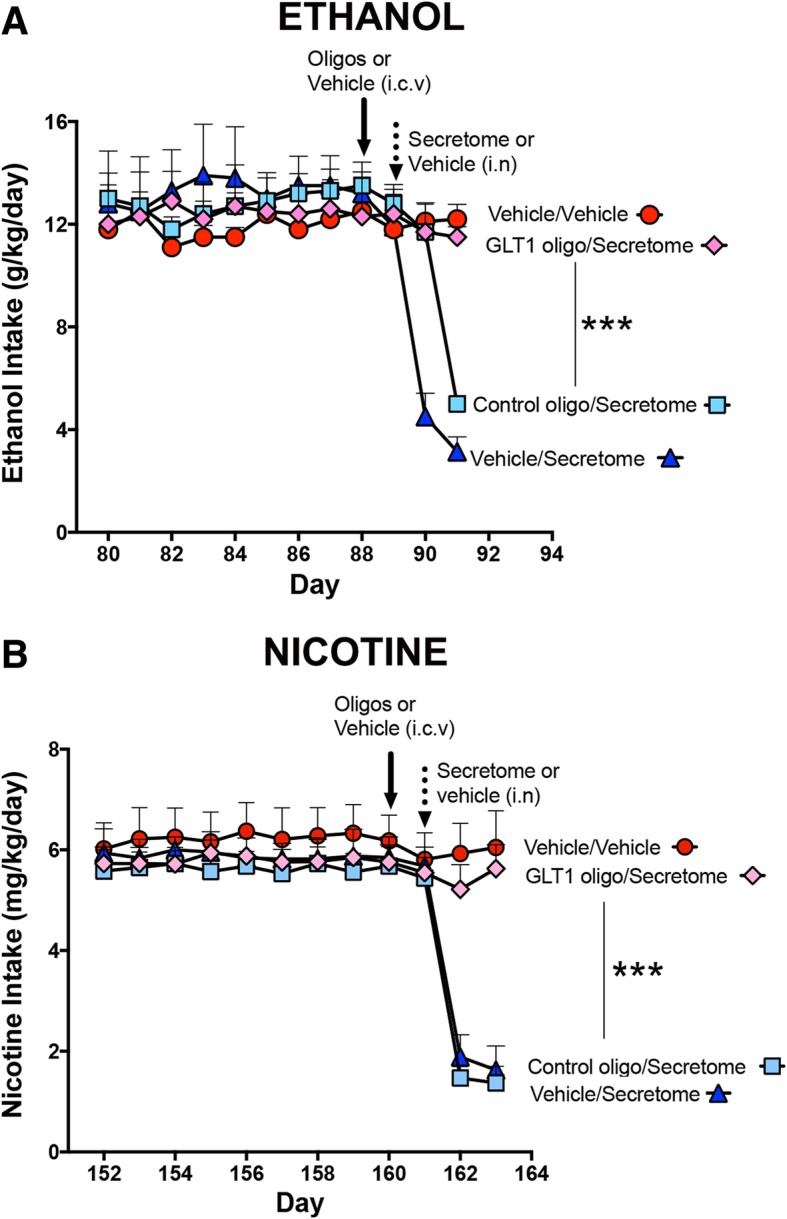
Antisense knockdown of GLT-1 prevented the inhibition of ethanol and nicotine intake induced by intranasal administration of adipose tissue-derived MSC secretome. **a** A single intranasal secretome administration to chronical ethanol-drinking rats that had received a prior lateral ventricle injection of either control antisense oligo (light blue squares) or vehicle (blue triangles) induced a 65% and 75% inhibition of ethanol intake, respectively, versus chronic ethanol-drinking rats treated with vehicle both intracerebrally and intranasally (red circles). Pretreatment with an anti-GLT-1 antisense oligo (pink diamonds) fully prevented the inhibition of ethanol intake induced by intranasal secretome. One-way ANOVA: *F*_3,12_ = 93, *p* < 0.0001; Bonferroni post-hoc: *p* < 0.001, GLT-1 oligo/secretome versus control oligo/secretome and versus vehicle/secretome; *p <* 0.001, vehicle/vehicle versus vehicle/secretome and versus control oligo/secretome. **b** A single intranasal secretome administration to chronical nicotine-drinking rats that had received a prior injection of control antisense oligo (light blue square) or vehicle (blue triangle) induced a 77% and 75% inhibition of nicotine intake, respectively, versus nicotine-drinking rats treated with vehicle both intracerebrally and intranasally (red circles). Pretreatment with the anti-GLT-1 antisense oligo (pink diamond) *fully* prevented the inhibition of nicotine intake induced by the subsequent intranasal secretome administration; one-way ANOVA: *F*_3,12_ = 23, *p* < 0.0001; Bonferroni post-hoc: *p* < 0.001, GLT-1 oligo/secretome versus control oligo/secretome and versus vehicle/secretome; *p <* 0.001, vehicle/vehicle versus control oligo/secretome and versus vehicle/secretome

As reported previously, the administration of exosomes derived from activated MSCs increased GLT-1 gene expression and temporarily inhibited chronic ethanol intake [10]. Further, the systemic administration of MSC spheroids (secretome “pumps”) to chronic ethanol-ingesting rats led to marked increases in the levels of the glutamate transporter GLT-1 in the nucleus accumbens and concomitantly inhibited chronic alcohol intake [9]. In a subsequent study, we examined the degree to which GLT-1 indeed mediates (rather than correlates with) the inhibitory effect of intranasal secretome both on chronic ethanol and chronic nicotine intakes. This was tested by GLT-1 knockdown by microinjection into the brain lateral ventricle of a specific anti-GLT-1 antisense oligonucleotide, prior to the intranasal administration of secretome derived from activated MSCs. Figure 8a shows that a single intranasal MSC secretome administration to animals that had ingested ethanol chronically and had received a prior injection (24 h before secretome) of control (inactive) oligo or vehicle reduced their chronic ethanol intake by 75% (*p* < 0.001), an inhibition that was *fully* prevented (*p* < 0.001) by the prior injection of the anti-GLT-1 antisense. For chronic nicotine intake (Fig. 8b), intranasal MSC secretome administration to animals that had received a prior injection of control oligo or vehicle inhibited nicotine intake by 80% (*p* < 0.001), an effect that was also *fully* prevented (*p* < 0.001) by the prior injection of the anti-GLT-1 antisense.

## Discussion

The present study shows that, as for other drugs of abuse [16–20, 47–49], chronic ethanol and chronic nicotine intakes lead to brain oxidative stress. The intranasal administration of antioxidant secretome derived from adipose tissue-derived activated MSCs inhibited chronic ethanol self-administration by 80% to 85% and virtually abolished the relapse binge drinking that followed a prolonged ethanol deprivation and a 60-min re-access to ethanol. Nearly identical results were obtained for nicotine intake following the intranasal administration of MSC-derived secretome. Thus, both chronic intake and relapse of two drugs of abuse were markedly inhibited by the intranasal administration of MSC-derived secretome.

The study shows that the brain oxidative stress (elevated GSSG/GSH ratio) induced by the chronic intake of both ethanol and nicotine was fully suppressed by the administration of MSC-derived secretome. A similar inhibition applied for neuroinflammation (astrocyte GFAP and microglia Iba-1 profiles). The question can be addressed as to which of these changes—oxidative stress or neuroinflammation—is directly related to the perpetuation of chronic ethanol or nicotine intake and is responsible for their relapse. Considering the existence of an oxidative stress/neuroinflammation self-perpetuating cycle [24–27], both the antioxidant and the anti-inflammatory activities of MSC-derived secretome likely contribute to reducing drug intake. Accordingly, the administration of the anti-inflammatory drug ibudilast was shown to inhibit chronic ethanol intake and relapse by 50%, both in rats and mice [50], while the oral administration of the potent antioxidant *N*-acetyl-cysteine reduced ethanol intake by 70 to 75% [11].

What is clear from the knockdown study is that the astrocyte GLT-1 transporter mediates the inhibitory effect of MSC secretome on chronic ethanol and nicotine self-administration, since the reduction in GLT-1 expression prevents the anti-addictive effects of the secretome. An unbiased brain transcriptome analysis of 6 strains/lines of rats (including the UChB rat used in these experiments) and mice bred for either high or low alcohol preference [51] showed that the differentially expressed genes between high and low drinking lines were primarily associated with glial (astrocytes and microglia) function, glial-neuronal communication, and energy metabolism (the main generator of reactive oxygen species), thus consistent with the present studies.

Glutamate homeostasis by astrocytes appears to play a major role in alcohol and other drug use disorders [28, 29, 43]. Several reports show that the astrocyte glutamate transporter GLT-1 activity is inhibited under oxidative stress conditions, both by oxidation of its sulfhydryl moieties [31] and by inactivation mediated by the lipid peroxidation product 4-HNE [32, 33]. As shown for chronic ethanol and nicotine studies, marked oxidative stress has also been reported for chronic cocaine administration [47]. Noteworthy, Reissner et al. [46] showed that the inhibition of cocaine relapse induced by the antioxidant *N*-acetyl cysteine was also prevented by GLT-1 transporter knockdown. Thus, the present study supports the view that glutamate overflow likely at the tripartite synapse in the nucleus accumbens [28] mediates the relapse of drugs of abuse.

## Conclusions

Overall, studies conducted show that the non-invasive intranasal administration of adipose tissue-derived activated mesenchymal stem cell secretome (i) markedly inhibited chronic intake and relapse of ethanol and nicotine, (ii) suppressed both the increased oxidative stress and neuroinflammation induced by chronic ethanol or nicotine intake, and (iii) demonstrate that the glutamate transporter GLT-1 mediates the inhibitory effect of the secretome on chronic ethanol and nicotine intake, possibly via a reduction of oxidative stress and neuroinflammation. Given the marked inhibitory effects and the duration of the effects of intranasally administered secretome on the self-administration and relapse of two drugs of abuse, possible translational implications are envisioned.

## Additional files

Additional file 1: **Figure S1.** Rats receiving daily nicotine dose (0.5 mg/kg, i.p.) for a 14-day period and subsequently allowed free-choice access between water and a nicotine solution displayed gradual increases of nicotine intake along with a gradual reduction of their water preference. (A) Naïve females UChB rats were intraperitoneally administered a daily dose of nicotine (0.5 mg/kg) for a 14-day period followed by free-choice access between water and nicotine (0.5 mg/ml to 40 mg/l) solution. Following the prolonged intake of the 40 mg/l nicotine solution, rats consumed a constant amount of 5.45 ± 0.10 mg of nicotine/kg/day (mean ± SEM), from day 53 to day 128 (each point is the average of 3 days of nicotine consumption, *n* = 26). (B) Increases in the intake of nicotine solutions were accompanied by reductions in their daily preference for water (water preference was calculated by dividing the daily water intake by total fluid consumption (total fluid intake = ml of water + ml of nicotine solution). Noteworthy, following the intraperitoneal injections of nicotine, there was already a partial preference for oral intake of the nicotine solution (5 mg/l) over the three initial days; which reduces the preference for water. (TIF 3115 kb)

Additional file 2: **Figure S2.** Intranasal administration of secretome, derived from adipose tissue-derived *non-activated* MSCs, inhibited only marginally and transiently chronic ethanol intake and did not prevent the alcohol relapse binge drinking behavior. (A, left) Two-way ANOVA of ethanol intake data obtained following 3 intranasal MSC secretome doses (160 μl containing 25 μg proteins derived from 1 × 10^6^ non-activated MSCs), given at weekly intervals (arrows), indicates significant effect of treatment (*F*_1,120_ = 108.6, *p* < 0.001), but not of day (*F*_1,120_ = 0.75, *p* = 0.71 N.S.), compared with the basal ethanol intake data obtained before treatment. Bonferroni post-hoc revealed that secretome derived from non-activated MSCs induced a 30% inhibition of ethanol intake compared with the basal levels of ethanol intake before the treatment (*p* < 0.05; *n* = 5 per group). (A, right) Chronic ethanol-drinking rats treated with five intranasal doses of secretome derived from non-activated MSCs consumed 1.80 ± 0.14 g ethanol/kg body weight during a 60-min ethanol re-access after 2 weeks of ethanol deprivation. (B) The blood ethanol levels determined immediately after the 60-min ethanol re-access were 100.0 ± 7.3 mg/dl, which is considered as “binge drinking” (> 80 mg/dl) suggesting that treatment with secretome derived from *non-activated* MSCs does not inhibit ethanol relapse. (TIF 4656 kb)

## Data Availability

All data generated or analyzed during this study are included in this published article and its supplementary information files.

## Abbreviations

4HNE: 4-Hydroxynonenal
BAC: Blood alcohol concentration
CYP2E1: Cytochrome P450 family 2 subfamily E member 1
GFAP: Glial fibrillary acidic protein
GLT-1: Glutamate transporter 1
GSH: Reduced glutathione
GSSG: Glutathione disulfide
Iba-1: Ionized calcium-binding adaptor molecule 1
ICV: Intracerebro-ventricular
IFN-γ: Interferon gamma
IκB: Inhibitor of kappa B
MSCs: Mesenchymal stem cells
NF-κB: Nuclear factor kappa-light-chain-enhancer of activated B cells
PBS: Phosphate-buffered saline
ROS: Reactive oxygen species
TNF-α: Tumor necrosis factor alpha
UChB: University of Chile Bibulous
α-MEM: Alpha minimal essential medium
