# Supplementary figures and images for: Intranasal mesenchymal stem cell secretome administration markedly inhibits alcohol and nicotine self-administration and blocks relapse-intake: mechanism and translational options
Source: Stem Cell Res Ther. 2019 Jul 8;10:205. doi: 10.1186/s13287-019-1304-z (PMC6615104; doi:10.1186/s13287-019-1304-z)

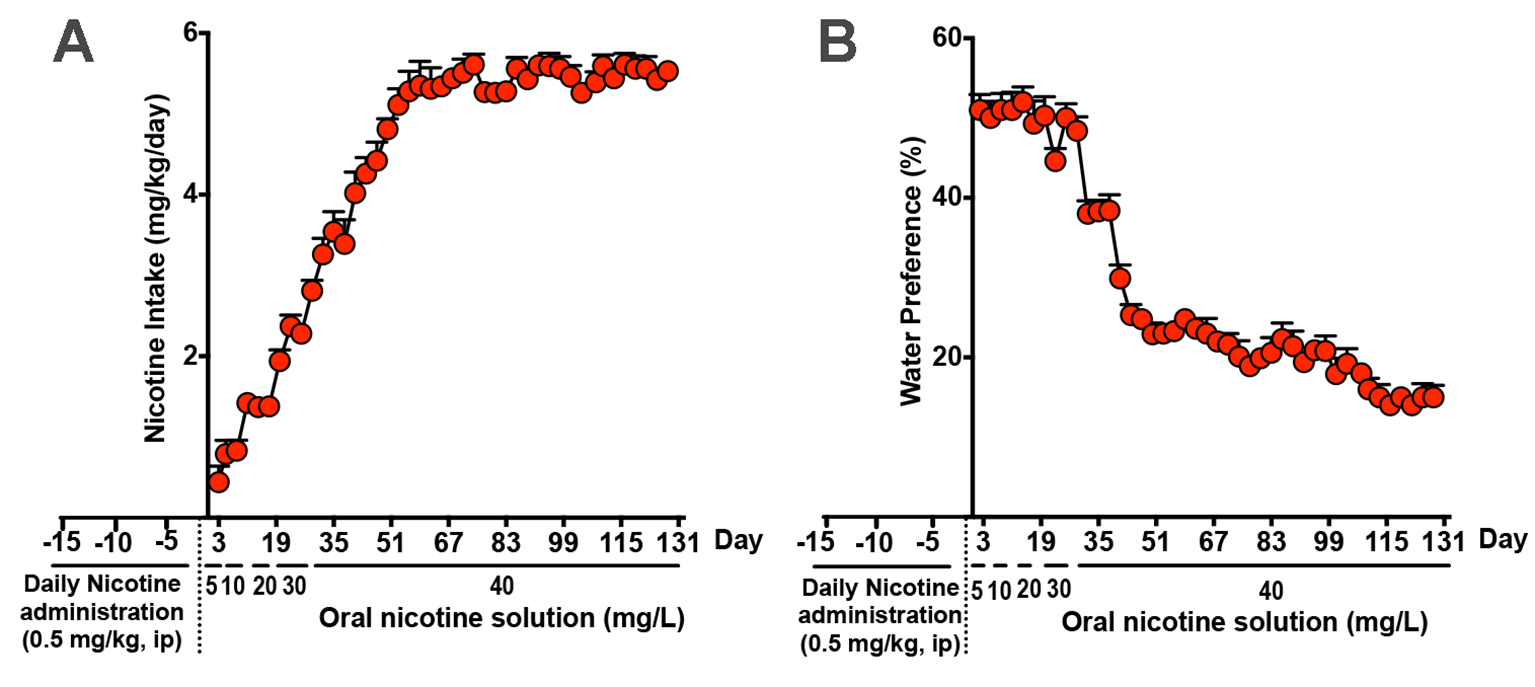

Supplement: Supplementary file 1 — Figure S1. Rats receiving daily nicotine dose (0.5 mg/kg, i.p.) for a 14-day period and subsequently allowed free-choice access between water and a nicotine solution displayed gradual increases of nicotine intake along with a gradual reduction of their water preference. (A) Naïve females UChB rats were intraperitoneally administered a daily dose of nicotine (0.5 mg/kg) for a 14-day period followed by free-choice access between water and nicotine (0.5 mg/ml to 40 mg/l) solution. Following the prolonged intake of the 40 mg/l nicotine solution, rats consumed a constant amount of 5.45 ± 0.10 mg of nicotine/kg/day (mean ± SEM), from day 53 to day 128 (each point is the average of 3 days of nicotine consumption, n = 26). (B) Increases in the intake of nicotine solutions were accompanied by reductions in their daily preference for water (water preference was calculated by dividing the daily water intake by total fluid consumption (total fluid intake = ml of water + ml of nicotine solution). Noteworthy, following the intraperitoneal injections of nicotine, there was already a partial preference for oral intake of the nicotine solution (5 mg/l) over the three initial days; which reduces the preference for water. (TIF 3115 kb) [file 13287_2019_1304_MOESM1_ESM.tif]

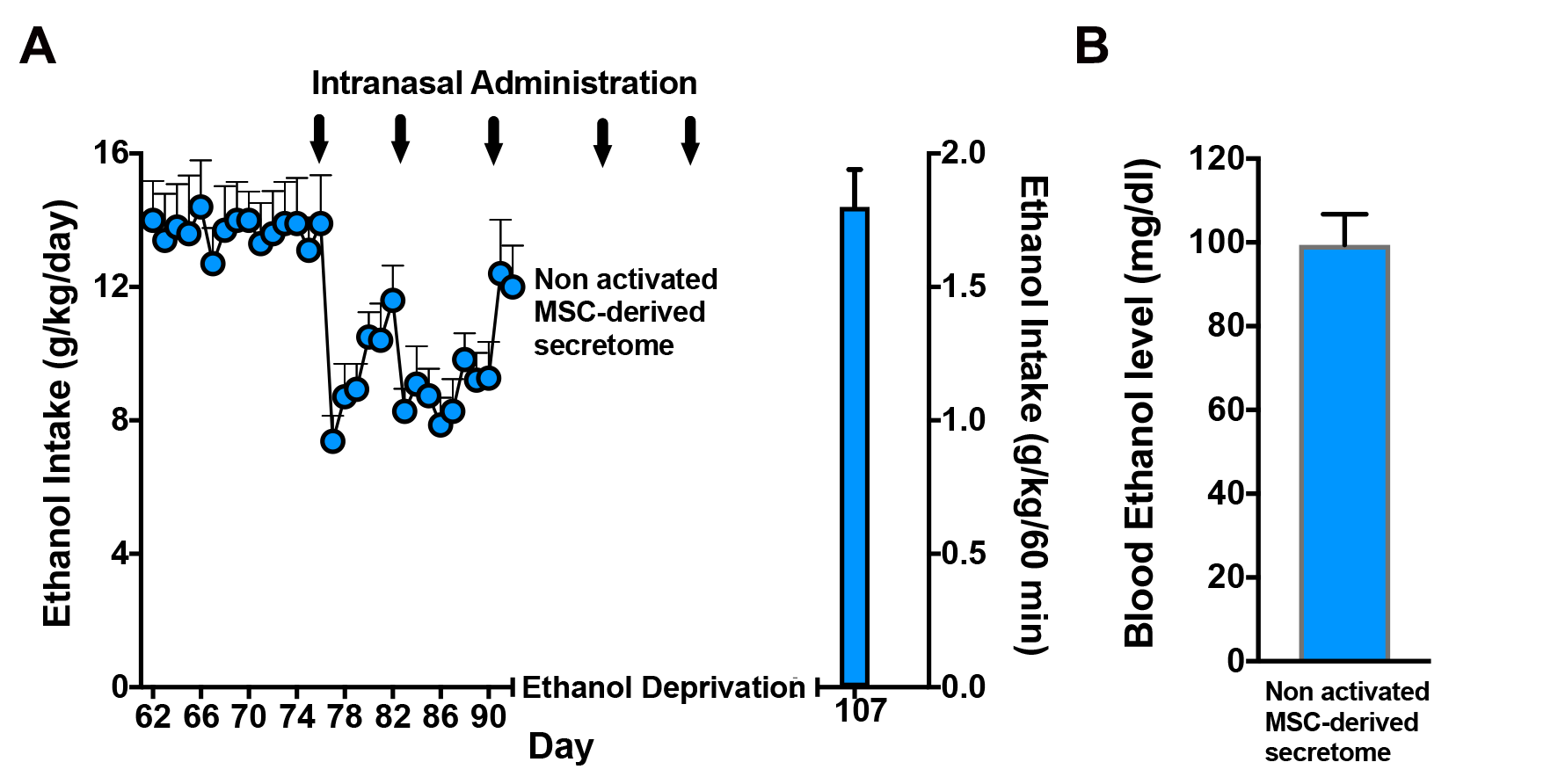

Supplement: Supplementary file 2 — Figure S2. Intranasal administration of secretome, derived from adipose tissue-derived non-activated MSCs, inhibited only marginally and transiently chronic ethanol intake and did not prevent the alcohol relapse binge drinking behavior. (A, left) Two-way ANOVA of ethanol intake data obtained following 3 intranasal MSC secretome doses (160 μl containing 25 μg proteins derived from 1 × 10^6^ non-activated MSCs), given at weekly intervals (arrows), indicates significant effect of treatment (F1,120 = 108.6, p < 0.001), but not of day (F1,120 = 0.75, p = 0.71 N.S.), compared with the basal ethanol intake data obtained before treatment. Bonferroni post-hoc revealed that secretome derived from non-activated MSCs induced a 30% inhibition of ethanol intake compared with the basal levels of ethanol intake before the treatment (p < 0.05; n = 5 per group). (A, right) Chronic ethanol-drinking rats treated with five intranasal doses of secretome derived from non-activated MSCs consumed 1.80 ± 0.14 g ethanol/kg body weight during a 60-min ethanol re-access after 2 weeks of ethanol deprivation. (B) The blood ethanol levels determined immediately after the 60-min ethanol re-access were 100.0 ± 7.3 mg/dl, which is considered as “binge drinking” (> 80 mg/dl) suggesting that treatment with secretome derived from non-activated MSCs does not inhibit ethanol relapse. (TIF 4656 kb) [file 13287_2019_1304_MOESM2_ESM.tif]
